# Analytical solution and optimal design for the output performance of Galfenol cantilever energy harvester considering electromechanical coupling effect

**DOI:** 10.1038/s41598-023-40111-x

**Published:** 2023-08-08

**Authors:** Lingzhi Wang, Chengling Lian, Dalin Shu, Zhitao Yan, Xiaochun Nie

**Affiliations:** School of Civil Engineering and Architecture, Chongqing University of Science and Technology, Chongqing, 401331 China

**Keywords:** Energy science and technology, Materials science, Mathematics and computing

## Abstract

The theoretical model of a Galfenol cantilever energy harvester is investigated for vibration energy harvesting. Compared with the numerical solution, the analytical solution can better capture the intrinsic effects of the physical parameters on the performance of the harvester. In this work, an electromechanical coupled distributed-parameter model of the Galfenol cantilever energy harvester is established based on Hamilton’s principle, linear constitutive equations of magnetostrictive material, and Faraday’s law of electromagnetic induction. The definitions and expressions of the electric damping and modified frequency are proposed due to the electromechanical coupling. The explicit analytical expressions of the average harvested power across the load resistance and tip vibration displacement of the Galfenol energy harvesting model are derived using the methods of Galliakin decomposition and electromechanical decoupling. The accuracy of the derived analytical results is verified by the experimental data and numerical solutions. The vibration response and energy harvesting performance of the Galfenol energy harvesting model are investigated by varying the excitation frequency, external resistance, and excitation acceleration amplitude. The analytical results show that, with the increase of the external load resistance and excitation frequency, the harvested power increases first and then decreases, indicating the existence of the optimal resistance and excitation frequency. From the explicit analytical expressions of the average harvested power, the optimal external load resistance or excitation frequency could be easily found to achieve the maximum harvested power for any fixed excitation frequency or external load resistance. The concept of proposing the electric damping and modified frequency for the Galfenol cantilever energy harvester simplifies the solution process for the output performances benefiting from the exact relationship between the output performances and the electromechanical coupling parameter derived in this work.

## Introduction

In order to alleviate the emerging energy crisis, green energy has gradually become the research focus of scholars. The existing green energy can be roughly divided into solar energy^1,2^, wind energy^3^, heat energy^4^ and environmental vibration energy^5,6^. In the field of ambient vibrational energy harvesting, the common energy harvesting mechanisms include electrostatic^7,8^, electromagnetic^9–11^, piezoelectric^12–14^ and magnetostrictive^15,16^. The electrical energy converted from the harvested ambient vibrational energy can be used to power self-powered devices, such as microelectromechanical devices, wireless sensor networks, embedded structural monitors and remote condition monitoring devices^17–19^.

Due to its merits of easy integration, small size, high energy density and no electromagnetic interference, the piezoelectric effect is widely used for vibration energy harvesting. The external excitation properties^13,19^ and external electrical circuits^20,21^ are the two prominent influences affecting the characteristics of piezoelectric energy harvesters. The detailed effects of the mentioned factors on the output performances for the piezoelectric energy harvesters have been analyzed comprehensively by numerical^3,22,23^, experimental^12,24,25^ and theoretical methods^17,24,26^ in the last two decades. It is noted that the precise relationship between the structural natural frequency, excitation frequency and external load resistance can only be derived by the theoretical method, thereby the electrical and mechanical optimizations and modulations for the coupling system could be undertaken simultaneously. In the relevant theoretical analysis, Khazaee et al.^26^ proposed an innovative approach to extract the damping coefficient only by the transient voltage responses, which provides interesting research directions for related future research. The unified electromechanical-coupled voltage equation considering the mechanical and electrical physics was derived in Khazaee’s work, of which, the fully coupled relationships between the voltage output, the driving vibration frequency, and the resistive electrical load are clarified. However, the application of piezoelectric technology is limited for the disadvantages of high cost, high brittleness, low tensile strength and easy aging.

Based on the Villari effect, magnetostrictive materials have become another smart material widely used in energy harvesting to convert mechanical energy into electrical energy^27,28^. As a new type of magnetostrictive material formed by adding the Ga element into the Fe element, Galfenol (Fe$_{100-x}$Ga$_x$ with $12<x < 30$) becomes a particularly promising transducer material. With the characteristics of high-efficient energy harvesting due to high magnetostriction, high permeability, small brittleness, high desirable tensile and compression mechanical, and wide working temperature range, Galfenol has been gradually applied to vibration energy harvesting in recent years^29,30^. In 2012, Rezaeealam et al.^31^ developed a numerical model to examine the performance of the vibration energy harvester with one-rod (unimorph) of Galfenol. The Armstrong model is employed in the static 3-D finite element model (FEM) of the energy harvester to consider the anisotropy of the Galfenol. Deng et al.^32^ experimentally investigated the output response of the Galfenol energy harvester under pulse excitation when the ratio of the thickness of the structure layer to the thickness of the Galfenol layer was 2. By a thorough experimental study, Apicella et al.^33^ investigated the effect of magnetic bias and the geometry of the physical structure on the output response by bonding one or more Galfenol sheets to an aluminum sheet. The experimental results showed that the harvested power reaches 40 mW when an acceleration amplitude of 4 g was adopted. Clemente et al.^34^ proposed a nonlinear equivalent circuit model for a multi-rod Galfenol energy harvester, the correctness of the equivalent circuit model under different resistances and pickup coils was verified through experiments and simulation analysis. Staruch et al.^35^ proposed a broadband magnetoelectric energy harvester consisting of both Galfenol and piezoelectric material. The harvester could operate in the non-resonant mode for broadband energy conversion using an induced rhombohedral to orthogonal phase transition in PIN-PMN-PT single crystal. Liu et al.^36^ developed an energy harvester with a Galfenol cantilever beam and designed an AC-DC converter with two working modes for a low-power harvester to promote the practicality of the vibration harvester. The experimental results showed that the 1 V AC voltage of the collector can be converted to 5 V DC voltage. Clemente et al.^37^ designed a force-driven kinetic energy harvester consisting of three Galfenol rods, and the experimental results of the proposed device was verified by nonlinear modeling with COMSOL Multiphysics software. Liu et al.^38^ established a rotating vibration energy harvesting device using Galfenol as the core material. It was used to convert the vibration energy generated by a moving vehicle into electrical energy. The experimental results revealed that the harvester could achieve an output voltage of 1.22 V at the acceleration amplitude of 9.6 g and the rotational speed of 90 r/min. A finite element numerical analysis of the Galfenol energy harvester was carried out by Ahmed et al.^39^ using the equivalent stress model. The Galfenol energy harvester numerical model was verified experimentally at different compressive loading cases ranging 20-80 MPa.

It is obvious that in the afore-mentioned studies, the experimental model or numerical simulation model was developed to analyze the performance of the Galfenol energy harvesters. In the theoretical modeling, Yoo et al.^40^ established the dynamic model of the Galfenol cantilever beam energy harvester based on the lumped parameter mechanical model of cantilever beam and the constitutive equation of magnetostrictive material. The power generation performance of the model was developed and tested. The maximum average output power of the model is 2.2 mW when the acceleration amplitude is 1 g and the frequency is 222 Hz. Cao et al.^41^ established the distributed parameter mechanical electric coupling dynamic model of the Galfenol cantilever beam vibration energy harvester using the Euler-Bernoulli beam theory, the constitutive equation of magnetostrictive materials, and Faraday’s law of electromagnetic induction. The vibration displacement and output harvested power of the model were numerically analyzed using the Runge-Kutta method. Cao et al.^42^ proposed a nonlinear coupled dynamic model of the Galfenol cantilever vibration energy harvester, which considered the change of the piezomagnetic coefficient of Galfenol with the stress and bias magnetic field. The output performances of the device were calculated numerically using the Newmark method. Xu et al.^43^ developed a hybrid energy harvester using the mechanisms of piezoelectric and magnetostrictive. The Galfenol alloy and nickel alloy were used as structural layers, the piezoelectric material was used as the additional layer. The calculated numerical solution of the theoretical model for the energy harvester was in good agreement with that of the experiment. To improve low-frequency broadband energy harvesting performances of the traditional cantilever energy harvester, Cao et al.^44^ designed a nonlinear Galfenol cantilever beam energy harvesting model with an elastic magnifier. The two-degree-of-freedom lumped parameter nonlinear coupled model of the proposed model was established and the frequency analytical expressions were derived. The time responses of the proposed harvester were obtained by Runge-Kutta method. Meng et al.^45^ combined the magneto-mechanical coupling model, the Galfenol constitutive equation, and the law of electromagnetic induction to establish the theoretical lumped parameter mechanical model of Galfenol energy harvester. The numerical results were compared to experimental harvested outputs of four prototype beams. Zhang et al.^46^ derived the dynamic equations of the composite cantilever energy harvester with Galfenol and a nonlinear energy sink, based on Newton’s second law, Hamilton’s principle and nonlinear boundary conditions. The displacement responses of the system were obtained by using the Runge-Kutta method and harmonic balance method. The results indicated that the proposed structure could achieve the desired effects of vibration reduction and energy harvesting.

In the above-mentioned literatures, the output performances of the Galfenol energy harvester were mostly obtained by laboratory tests and finite element software simulations. While in the analysis of the theoretical model for the Galfenol energy harvester, the output performances were basically calculated using numerical methods, such as Runge-Kutta method and Newmark method. It is difficult for them to capture the intrinsic effects of the physical parameters of the Galfenol energy harvester on the performance and optimize analysis and design the harvester. In this work, the electromechanical coupled distributed-parameter model of the Galfenol cantilever energy harvester is proposed based on Hamiltons principle, linear constitutive equations of magnetostrictive materials, and Faradays law of electromagnetic induction. The definitions and expressions of the electric damping and modified frequency are proposed due to the electromechanical coupling, and the explicit analytical expressions of the output performances for the energy harvesting model are derived. In addition, the effects of external excitation acceleration amplitude, excitation frequency, and external load resistance on the output performances of the energy harvesting model are studied analytically. Finally, the energy harversting capability of the proposed harvester is optimized from the aspects of external load resistance and excitation frequency.

## Mathematical model

### Electromechanical coupled distributed parameter model

**Figure 1:**
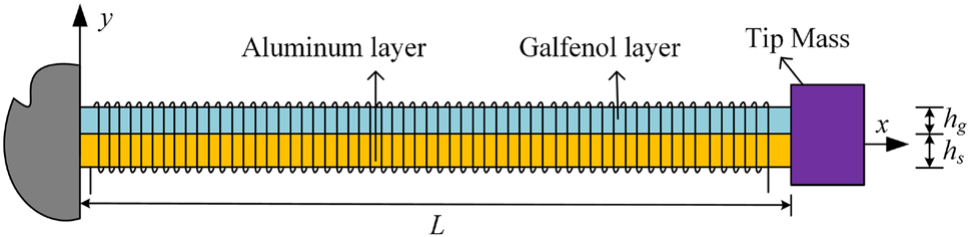
Schematic diagram of the Galfenol cantileve energy harvester.

As shown in Fig. 1, the Galfenol energy harvester consists of a Galfenol additional layer and an aluminum structural layer. The length and width of the two material layers are both *L* and *b*, the thickness of the Galfenol layer and the aluminum layer are respectively $h_g$ and $h_s$. A lumped mass $M_t$ is placed at the free end of the cantilever beam structure. Let *x*-axis and *y*-axis are respectively the length direction and vertical direction of the beam, then the *XY*-plane is the neutral plane of the beam. When the cantilever beam structure vibrates up and down along the *y*-axis, the magnetic induction intensity in the Galfenol layer will change due to the presence of the inverse magnetostriction effect. Then an induced voltage will be generated at both ends of the coil, and electrical energy is generated according to Faraday’s law of electromagnetic induction. Therefore, by using the proposed energy harvester, the waste vibration energy in the environment can be converted into green electrical energy, providing energy for low-power electronic devices.

Assuming the electromagnetic coil is long enough to ignore the edge effect, the magnetic field strength H applied along the longitudinal axis of the Galfenol layer can be expressed by Ampere’s law^46^ as

1 $$\begin{aligned}  {H_0} = N \cdot {I_g}\left( t \right) /L \end{aligned}$$

Where ${I_g}\left( t \right)$ is the induced current in the coil, and *N* is the number of turns of the coil.

The stress-strain of the structural aluminum layer conforms to Hooke’s law, i.e., ${\sigma _s} = {E_s}{\varepsilon _s}$, ${E_s}$ and ${\varepsilon _s}$ are respectively the Young’s modulus and strain of the aluminum layer. The linear constitutive equation of the Galfenol layer^47^ is adopted as

2 $$\begin{aligned} { \varepsilon _g} &= {\sigma _g}/{E_g} + {d_0} \cdot {H_0}\\ B &= {d_0} \cdot {\sigma _g} + \mu \cdot {H_0} \end{aligned} $$

Where ${\sigma _g}$ is the axial stress of the Galfenol layer along the x-axis, $\mu$ is the material permeability of the Galfenol layer, $d_0$ is the magneto-mechanical coupling piezomagnetic coefficient of the Galfenol layer, $E_g$ is the Young’s modulus of the Galfenol layer, and *B* is the magnetic induction strength of the Galfenol layer.

According to the Euler Bernoulli beam assumption and the extend Hamilton’s principle^3,12^, the variation of the total potential energy of the cantilevered beam Galfenol energy harvester model can be obtained as

3 $$\begin{aligned} \int _{{t_1}}^{{t_2}} {\left( {\delta T - \delta V + \delta {W_{nc}}} \right) } dt = 0 \end{aligned} $$

where *T*, *V*, and $W_{nc}$ are respectively the kinetic energy, potential energy, and virtual work due to the nonconservative forces of the energy harvester model.

The variation of kinetic energy $\int _{{t_1}}^{{t_2}} {\delta T} dt$ can be expressed as

4 $$\begin{aligned}  \int _{{t_1}}^{{t_2}} {\delta T} dt & = \int _{{t_1}}^{{t_2}} {\left[ {\frac{1}{2}\delta \int _0^L m {{\left( {\frac{{\partial {w_{rel}}\left( {x,t} \right) }}{{\partial t}} + \frac{{\partial {w_b}\left( t \right) }}{{\partial t}}} \right) }^2} dx} \right. } \\ & \quad \left. { + \frac{1}{2}\delta {M_t}{{\left( {\frac{{\partial {w_{rel}}\left( {L,t} \right) }}{{\partial t}} + \frac{{\partial {w_b}\left( t \right) }}{{\partial t}}} \right) }^2} + \frac{1}{2}\delta {I_t}{{\left( {\frac{{{\partial ^2}{w_{rel}}\left( {L,t} \right) }}{{\partial x\partial t}}} \right) }^2}} \right] dt \end{aligned}$$

where ${w_{rel}}\left( {x,t} \right)$ is the transverse vibration displacement relative to the fixed end of the energy harvester model, ${w_b}\left( {x,t} \right)$ is the transverse vibration displacement of the fixed end, *m* is the mass per unit length of the composite beam of the energy harvester model, $M_t$ and $I_t$ are the mass and rotational inertia of the lumped mass at the free end of the energy harvester model.

The variation of potential energy $\int _{{t_1}}^{{t_2}} {\delta V} dt$ is given by

5 $$\begin{aligned} \int _{{t_1}}^{{t_2}} {\delta V} dt = \int _{{t_1}}^{{t_2}} {\int _0^L {EI\frac{{{\partial ^2}{w_{rel}}\left( {x,t} \right) }}{{\partial {x^2}}}\frac{{{\partial ^2}\delta {w_{rel}}\left( {x,t} \right) }}{{\partial {x^2}}}dx} } dt \end{aligned}$$

where *EI* is the flexural stiffness of the composite cantilever beam.

The nonconservative forces include the electromagnetic force, damping force. Therefore, the variation of virtual work variables $\int _{{t_1}}^{{t_2}} {\delta {W_{nc}}} dt$ due to the nonconservative forces can be written as

6 $$\begin{aligned} \int _{{t_1}}^{{t_2}} {\delta {W_{nc}}} dt = \int _{{t_1}}^{{t_2}} {\delta {W_{ele}}} dt + \int _{{t_1}}^{{t_2}} {\delta {W_{damp}}} dt \end{aligned} $$

where ${W_{ele}}$ and ${W_{damp}}$ are the virtual work due to electromagnetic and damping forces, respectively. The variation of virtual work due to electromagnetic force is given by

7 $$\begin{aligned} \int _{{t_1}}^{{t_2}} {\delta {W_{ele}}} dt = - \int _{{t_1}}^{{t_2}} {\int _0^L {{M_{ele}}\frac{{{\partial ^2}\delta {w_{rel}}(x,t)}}{{\partial {x^2}}}} dx} dt \end{aligned}$$

where ${M_{ele}}$ is the moment caused by the magnetostrictive effect and is expressed as

8 $$\begin{aligned} {M_{ele}} = - \frac{1}{2}{E_g}b{d_0}\left( {N{I_g}\left( t \right) /L} \right) {\left( {{h_c} - {h_b}} \right) ^2}\left[ {H\left( x \right) - H\left( {x - L} \right) } \right] \end{aligned} $$

where $h_c$ and $h_b$ are respectively the distance from the top and bottom of the Galfenol layer to the neutral layer of the composite beam, $H\left( x\right)$ is the Heaviside step function. Based on the proportional damping assumptions, the virtual work due to the damping force can be represented as

9 $$\begin{aligned} \int _{{t_1}}^{{t_2}} {\delta {W_{damp}}} dt = - \int _{{t_1}}^{{t_2}} {\int _0^L {{c_s}I \cdot \frac{{{\partial ^5}\delta {w_{rel}}(x,t)}}{{\partial {x^4}\partial t}}} dx - \int _0^L {{c_a}} \cdot \frac{{\partial \delta {{({w_{rel}}(x,t) + {w_b}(t))}^2}}}{{\partial t}}dx} dt \end{aligned}$$

where *I* is the cross-sectional area moment of the composite beam, $c_s$ and $c_a$ are respectively the strain rate damping coefficient and viscous air damping coefficient of the cantilever beam.

Substituting Eqs. (4), (5) and (6) into Eq. (3), the governing equation for the Galfenol cantilever energy harvester is obtained as

10 $$\begin{aligned} &m\frac{{{\partial ^2}{w_{rel}}\left( {x,t} \right) }}{{\partial {t^2}}} + EI\frac{{{\partial ^4}{w_{rel}}\left( {x,t} \right) }}{{\partial {x^4}}} + {c_s}I \cdot \frac{{{\partial ^5}{w_{rel}}(x,t)}}{{\partial {x^4}\partial t}} + {c_a}\frac{{\partial {w_{rel}}(x,t)}}{{\partial t}} + \frac{{{\vartheta _\mathrm{{g}}}N{I_g}\left( t \right) }}{L}\frac{{{\partial ^2}\left[ {H\left( x \right) - H\left( {x - L} \right) } \right] }}{{\partial {x^2}}}\\&= - \mathrm{{(}}{M_t}\delta (x - L) + m)\frac{{{\partial ^2}{w_b}\left( t \right) }}{{\partial {t^2}}} - {c_a}\frac{{\partial {w_b}(t)}}{{\partial t}} \end{aligned} $$

where $\delta (x)$ is Delta function, and the Galfenol coupling term ${\vartheta _g}$ is defined as ${\vartheta _g} = - {E_g}b{d_0}\frac{1}{2}{\left( {{h_c} - {h_b}} \right) ^2}$.

Based on Faraday’s law of electromagnetic induction^40^, the induced voltage generated in Galfenol layer of length $\Delta L$ can be expressed as

11 $$\begin{aligned}  \Delta {V_g}(t) = - \Delta L\frac{{NA}}{L}\frac{{dB}}{{dt}} \end{aligned} $$

where *A* is the cross-sectional area of the coil given by $A \approx b\left( {{h_s} + {h_g}} \right)$, $\Delta {V_g}(t) \rightarrow d{V_g}(t)$ and $\Delta L \rightarrow dx$ . Substituting Eqs. (1) and (2) into Eq. (11) and integrating the resulting equation over the length *L* yields the total induced voltage from the coil

12 $$\begin{aligned} {V_g}\left( t \right) = - \frac{{N{d_0}{E_g}A}}{L}\int _0^L {\frac{{d{\varepsilon _g}}}{{dt}}} dx - \frac{{{L_0}}}{R}\frac{{d{V_g}\left( t \right) }}{{dt}} \end{aligned} $$

where ${L_0} = (\mu - {d_0}^2{E_g}){N^2}A/L$ is the coil’s equivalent inductance, *R* is the sum of the internal resistance of the coil $R_C$ and the external load resistance $R_L$.

Substituting the strain calculation formula into Eq. (12) yields

13 $$\begin{aligned} {V_g}\left( t \right) = G\int _{{L_1}}^{{L_2}} {\frac{{{d^3}{w_{rel}}(x,t)}}{{d{x^2}dt}}} dx - \frac{{{L_0}}}{R}\frac{{d{V_g}\left( t \right) }}{{dt}} \end{aligned} $$

where $G\mathrm{{ = }}\frac{{N{d_0}{E_g}A{h_{gc}}}}{L}$ is the electromechanical coupling coefficient of the Galfenol cantilever beam, and $h_{gc}$ is the distance from the center layer of the Galfenol to the neutral layer of the composite beam in the thickness direction.

### Electromechanical coupled reduced order model

Galerkin decomposition method^48^ is adopted to discretize the transverse displacement of the cantilever beam ${w_{rel}}(x,t)$ into spatial and time variables as the following relation

14 $$\begin{aligned} {w_{rel}}(x,t) = \sum \limits _{r = 1}^\infty {{\phi _r}(x)q{}_r(t)} \end{aligned} $$

where ${\phi _r}(x)$ and $q{}_r(t)$ are the *r* th mode shape and modal coordinates of the cantilever beam Galfenol energy harvester, respectively.

According to the previous work^3,49^, the second or higher natural frequency of the cantilever beam is much larger than the first one. The responses of the one-mode and three-mode responses of the system were compared by Bibo^50^, and a conclusion is drawn that the single-mode was sufficient to capture the system’s response. This conclusion has been proved to be true and adopted in many practical applications and researches^17,24,26,51^. Thus, only the first mode is considered in this paper to simplify the simulation. Substituting Eq. (14) into the governing Eqs. (10) and (13), and then using orthogonal and boundary conditions, the reduced governing equations of the Galfenol cantilever energy harvester are derived as

15 $$\begin{aligned} &\ddot{q}\left( t \right) + {C_m}\dot{q}\left( t \right) + \omega _{}^2q\left( t \right) \mathrm{{ + }}\frac{{{\theta _g}}}{R}{V_g}\left( t \right) = F\left( t \right) \end{aligned} $$

16 $$\begin{aligned} &\frac{{{L_0}}}{R}{\dot{V}_g}\left( t \right) + {V_g}\left( t \right) - {\theta _g}\dot{q}\left( t \right) = 0 \end{aligned} $$

where ${\theta _g}\mathrm{{ = }}\frac{{N{d_0}{E_g}{h_\mathrm{{g}}}{b_\mathrm{{g}}}{h_{gc}}{\phi ^\prime }\left( L \right) }}{L}$, ${C_m} = 2\xi \omega$, $\xi$ and $\omega$ are respectively the mechanical damping ratio and first natural frequency of the composite cantilever beam.

When the external excitation is a harmonic excitation with the frequency of $\omega _b$, as a typical way of dealing with these coupled series of equations, the input and output steady-state solutions of the energy harvester can be written in the following form^26,52,53^

17 $$\begin{aligned} F\left( t \right) & = {F_0}\cos ({\omega _b}t + \alpha )\\ q\left( t \right) & = {q_0}\mathrm{{cos}}({\omega _b}t)\\ {V_g}\left( t \right) & = {V_0}\mathrm{{cos}}({\omega _b}t + \varphi ) \end{aligned}$$

where $F_0$ is the modal force amplitude and expressed as ${F_0}\mathrm{{ = }}\left[ { - m\int _0^L {\phi (x)} dx - {M_t}\phi (L)} \right] {a_0}$, $a_0$, $q_0$ and $V_0$ are respectively the amplitude of acceleration, modal coordinate and voltage, $\alpha$ is the phase angle between $F\left( t \right)$ and $q\left( t \right)$, and $\varphi$ is the phase angle between ${V_g}\left( t \right)$ and $q\left( t \right)$ .

According to the electromechanical decoupled method^14,24,26^, the voltage amplitude $V_0$ can be obtained by substituting Eq. (17) into Eq. (16) as the function of the modal coordinates amplitude $q_0$ and given by

18 $$\begin{aligned} {V_0} = \frac{{{\theta _g}{\omega _b}}}{{\sqrt{1 + C_g^2\omega _b^2} }}{q_0} \end{aligned} $$

where $${C_g}= \frac{{{L_0}}}{R}$$.

Substituting the expression of voltage into Eq. (15), we obtain the decoupled model for the Galfenol energy harvester as

19 $$\begin{aligned} \ddot{q}\left( t \right) + ({C_m} + {C_e})\dot{q}\left( t \right) + {\bar{\Omega }^2}q\left( t \right) = F\left( t \right) \end{aligned} $$

where $C_e$ and $\bar{\Omega }$ are the electrical damping and the modified frequency corresponding to the base excitation whose expressions are ${C_e} = \frac{{\theta _g^2}}{{R\left( {1 + C_g^2{\omega _b}^2} \right) }}$ and $\bar{\Omega }\mathrm{{ = }}\sqrt{{\omega ^2} + \frac{{{C_g}\theta _g^2{\omega _b}^2}}{{R\left( {1 + C_g^2{\omega _b}^2} \right) }}}$, respectively. Obviously, the electrical damping $C_e$ and the modified frequency $\bar{\Omega }$ are composite functions of the external excitation frequency $\omega _b$ and the external load resistance $R_L$, and represent the electromechanical coupling effects of the coupled system, which is similar to the well-known fact for piezoelectric energy harvester that the modified natural frequency are electrical-load and excitation frequency dependent^13,26^.

Multiplying Eq. (19) by $\dot{q}\left( t \right)$ and integrating the resulting equation from $t_1$ to $t_2$ , where ${t_2} - {t_1}\mathrm{{ = }}{\pi \mathord {{/}} {{\omega _b}}}$ , the first relationship between $F_0$ and $q_0$ is obtained as

20 $$\begin{aligned} {F_0}\sin (\alpha )  = \left( {{C_m} + {C_e}} \right) {\omega _b}{q_0} \end{aligned} $$

To obtain the second relationship between $F_0$ and $q_0$ , we differentiate Eq. (19) with respect to time *t* and multiply the resulting equation by $\dot{q}\left( t \right)$. By integrating each term from $t_1$ to $t_2$ , yields

21 $$\begin{aligned} {F_0}\cos (\alpha ) = ({\bar{\Omega }^2} - \omega _b^2){q_0} \end{aligned} $$

Eliminating the phase angle $\alpha$ from Eqs. (20) and (21), the amplitude of the modal coordinate $q_0$ is calculated as

22 $$\begin{aligned} {q_0} = \frac{{{F_0}}}{{\sqrt{{{\left[ {({\bar{\Omega }^2} - \omega _b^2)} \right] }^2} + {{\left( {\left( {{C_m} + {C_e}} \right) {\omega _b}} \right) }^2}} }} \end{aligned} $$

The averaged harvested power across the external load resistance $R_L$ is given by $P_L^{av} = \frac{1}{T}\int _0^T {{{\left( {\frac{{{V_g}\left( t \right) }}{R}} \right) }^2}{R_L}} dt = \frac{{V_0^2{R_L}}}{{2{R^2}}}$ , where *T* is the period of the harvesting system. Substituting the Eq. (18) into the expression of $P_L^{av}$, the external average harvested power is then obtained as

23 $$\begin{aligned} P_L^{av} = \frac{{{R_L}{C_e}\omega _b^2q_0^2}}{{2R}} \end{aligned} $$

The amplitude of the tip displacement and average harvested power for the Galfenol energy harvester are calculated from $q_0$ using ${A_{tip}} = \phi \left( L \right) {q_0}$ and Eq. (23) as below

24 $$\begin{aligned} {A_{tip}} = \frac{{\phi \left( L \right) {F_0}}}{{\sqrt{{{\left[ {({\bar{\Omega }^2} - \omega _b^2)} \right] }^2} + {{\left( {\left( {{C_m} + {C_e}} \right) {\omega _b}} \right) }^2}} }} \end{aligned} $$

25 $$\begin{aligned} P_L^{av} = \frac{{{R_L}{C_e}\omega _b^2F_0^2}}{{2[{{({\bar{\Omega }^2} - \omega _b^2)}^2} + {{\left[ {\left( {{C_m} + {C_e}} \right) {\omega _b}} \right] }^2}]R}} \end{aligned} $$

As can be seen from Eqs. (24) and (25), the amplitude of the tip displacement and average harvested power for the Galfenol energy harvester are both excitation frequency and external load resistance dependent, as well as the electrical damping $C_e$ and the modified frequency $\bar{\Omega }$. These relations can be effectively used to provide an estimate and optimize the performance of cantilever-based energy harvesters without the need to simultaneously solve the coupled equations or test a system over a wide range of electric loads. In addition, similar to the damping determination method proposed by Khazaee^26^, the structural damping $C_m$ and structural natural frequency $\omega$ can be determined in reverse from Eq. (24) or (25) at any fixed excitation frequency and external load resistance.

From the expressions of the tip displacement amplitude and average harvested power given by Eqs. (24) and (25), it can be seen that the average output power across the external resistance is related to both the excitation frequency $\omega _b$ and external load resistance value $R_L$.

#### Optimal design

For the optimal design of the Galfenol energy harvester, we first consider that when the excitation frequency $\omega _b$ of the external load is determined, let $\frac{{\partial P_L^{av}}}{{\partial {R_L}}} = 0$ yields

26 $$\begin{aligned} {\bar{A}_1}{R^2} + {\bar{C}_1} = 0 \end{aligned} $$

where $R=R_C+R_L$, ${\bar{A}_1}\mathrm{{ = }} - \omega _b^2(C_m^2 - 2{\omega ^2}) + {\omega ^4} + \omega _b^4$ and ${\bar{C}_1}\mathrm{{ = }}L_0^2\omega _b^6 + \omega _b^4\left[ {{L_0}\left( {C_m^2{L_0} - 2{L_0}{\omega ^2} - 2\theta _g^2} \right) + R_C^2} \right] + \omega _b^2\left[ {{{\left( {{L_0}{\omega ^2} + \theta _g^2} \right) }^2}} \right.$ $\left. { + R_C^2\left( {C_m^2 - 2{\omega ^2}} \right) + 2{C_m}{R_C}\theta _g^2} \right] + R_C^2{\omega ^4}$. Thus, the optimal external load resistance $R_L^{opt}$ can be obtained from Eq. (26) by finding the positive real roots.

When the external load resistance value $R_L$ is determined, in order to obtain the maximum power, let $\frac{{\partial P_L^{av}}}{{\partial {\omega _b}}} = 0$ , then we have

27 $$\begin{aligned} {\bar{A}_2}\omega _b^6 + {\bar{B}_2}\omega _b^4 + {\bar{C}_2} = 0 \end{aligned} $$

where ${\bar{A}_2}\mathrm{{ = }}2L_0^2$, ${\bar{B}_2}\mathrm{{ = }}{\left( {{R_L} + {R_C}} \right) ^2} + {L_0}\left( { - 2\theta _g^2 + {L_0}\left( {C_m^2 - 2{\omega ^2}} \right) } \right)$ and ${\bar{C}_2}\mathrm{{ = }} - {\left( {{R_L} + {R_C}} \right) ^2}{\omega ^4}$. That is, the optimal excitation frequency $\omega _b^{opt}$ can be calculated from Eq. (27) by finding the positive real roots.

## Results and discussion

### Verification of analytical solutions

In order to verify the accuracy of the proposed theoretical model and analytical derivation, the analytical results calculated directly by the Eq. (25) derived in this paper are compared with the experimental data^40^ and numerical solutions^41^. The physical and geometric properties of the composite cantilever beam used in this paper are consistent with the relevant parameters in references^40,41^, as shown in Table 1. The first-order natural frequency $f_0$ given by ${f_0} = {\omega \mathord {{/}} {2\pi }}$ of the energy harvester is 224.0 Hz.

**Table 1:** Physical and geometric properties of the Galfenol cantilever energy harvester.

Parameter	Description	Value
*L*	Length of the aluminum layer and Galfenol layer(mm)	38
*b*	Width of the aluminum layer and Galfenol layer (mm)	6.35
$h_{s}$	Thickness of the aluminum layer and (mm)	1.27
$h_{g}$	Thickness of the Galfenol layer (mm)	0.76
$E_s$	Young’s modulus of the aluminum layer (GN/m$^2$)	68
$E_g$	Young’s modulus of the Galfenol layer (GN/m$^2$)	70
$\rho _{s}$	Density of the aluminum layer (kg/m$^3$)	2700
$\rho _{g}$	Density of the Galfenol layer (kg/m$^3$)	7496
$\xi$	Mechanical damping ratio of the first modal	0.014
$M_t$	Mass of the tip lumped mass (g)	8.1
$\mu$	magnetic permeability(H/m)	$920\pi \times 10^{-7}$
$d_0$	Piezomagnetic coefficient (T/Gpa)	34
$R_C$	Internal resistance of the coil ($\Omega$)	36.4

**Figure 2:**
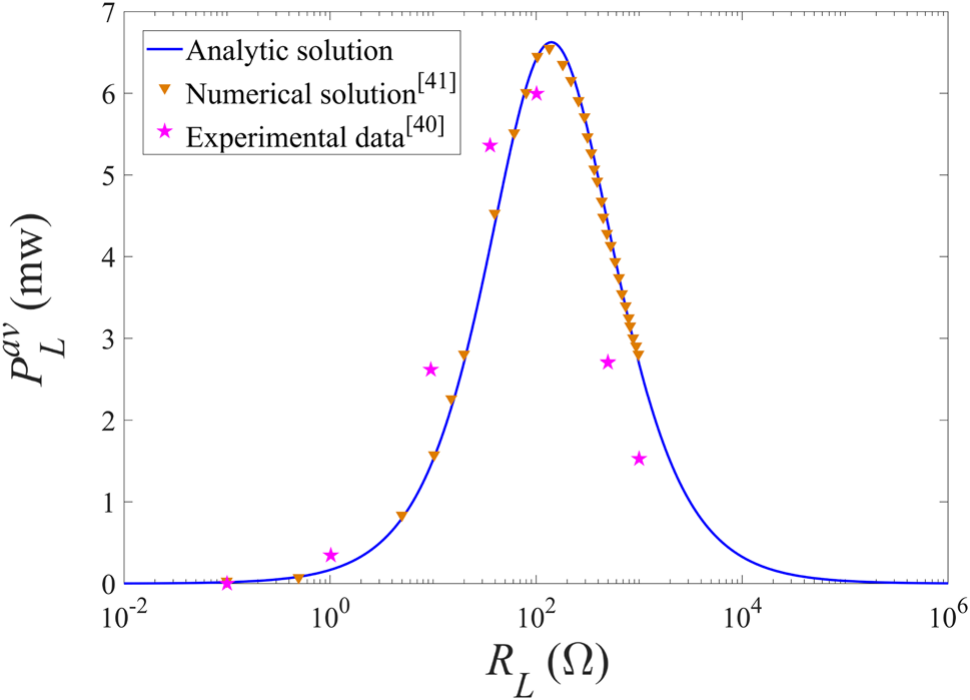
Variations of the average harvested power with the external resistance for different acceleration amplitudes.

The variation of the average harvested power for the Galfenol energy harvester obtained by the three methods mentioned above with the external resistance are presented in Fig. 2, at the excitation frequency of 222 Hz and the acceleration amplitude of 2g. As can be seen from the Fig. 2, the theoretical analytical results are in good agreement with the numerical solutions of the reference^41^ and are basically consistent with the experimental data in the reference^40^. The error between the analytical solution and the experimental data may be due to the ignoring of the nonlinear characteristics of the piezomagnetic coefficient for the Galfenol layer, as reported in the previous researches^41,42^. In general, the proposed theoretical model and analytical solution are reliable.

#### Parametric analysis

#### Effects of external resistance and excitation frequency on electrical damping and modified frequency

**Figure 3:**
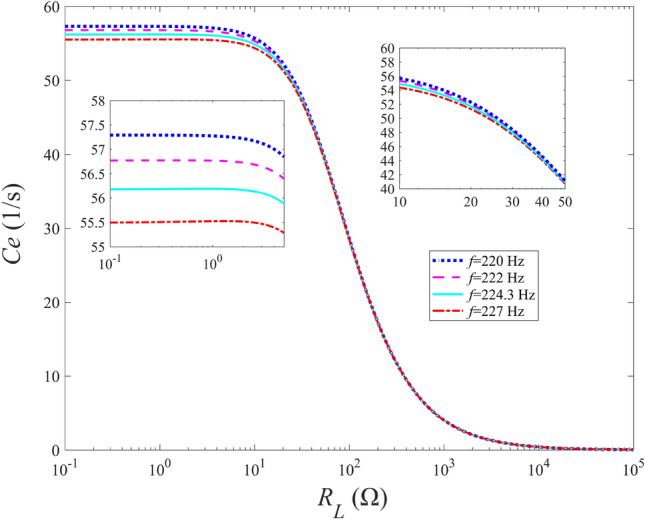
Variations of electrical damping with external resistance for different excitation frequencies.

Figure 3 shows the variations of the electrical damping of the energy harvester with the external resistance when the excitation frequencies ($f = {{{\omega _b}} \ {2\pi }}$) is fixed at 220 Hz, 222 Hz, 224.3 Hz and 227 Hz respectively. As shown in Fig. 3, the electric damping decreases as the excitation frequency increases. Besides, for the four adopted excitation frequencies, the electric damping remains basically unchanged at ${R_L} < 2$ $\Omega$, and declines sharply at 2 $\Omega \le {R_L} \le {10^4}$ $\Omega$, then almost close to 0 at ${R_L} > {10^4}$ $\Omega$ . When the external resistance exceeds 50 $\Omega$, the electric damping for the energy harvester with different excitation frequencies are almost the same. Generally speaking, the electrical damping exhibits a strong dependence on the load resistance in the range between 2 $\Omega$ and $10^4$ $\Omega$, and the sensitivity of the electric damping to the load resistance is weak when the load resistance is beyond the abovementioned range. When the load resistance exceeds ${10^4}\Omega$, the electric damping generated by the electromechanical coupling effect is almost equal to 0, which presents a negligible dependency on the load resistance and excitation frequency.

**Figure 4:**
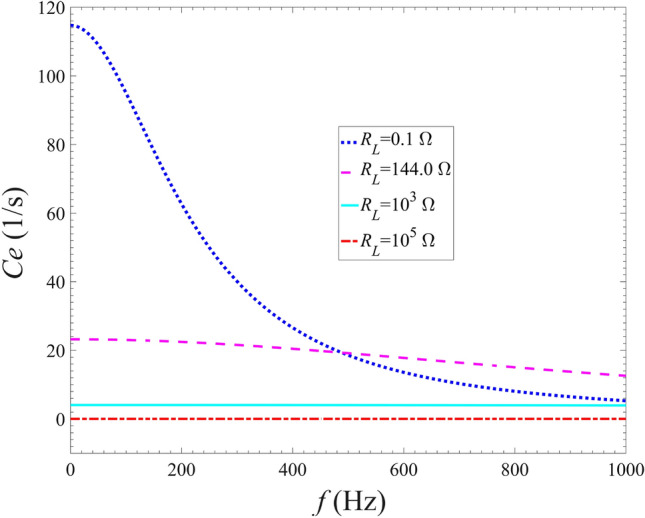
Variations of the electrical damping of the energy harvester with the excitation frequency for different external load resistances.

The variation of the electrical damping of the energy harvester with the increasing of the excitation frequency with different external resistances is shown in Fig. 4. The results show that when the external resistance $R_L$ is set as 0.1 $\Omega$, the electrical damping gradually decreases as the excitation frequency increases. For the case of ${R_L} = 144.0$ $\Omega$, the electric damping decreases slightly as the excitation frequency increases. With the increase of the excitation frequency, the electric damping remains basically unchanged for ${R_L} = {10^3}$ $\Omega$ and ${R_L} = {10^3}$ $\Omega$. It indicates that when the resistance is greater than $10^3$ $\Omega$, the sensitivity of the electric damping to the excitation frequency and the electromechanical coupling effects are both weak, which is corresponding to the results presented in Fig. 3.

**Figure 5:**
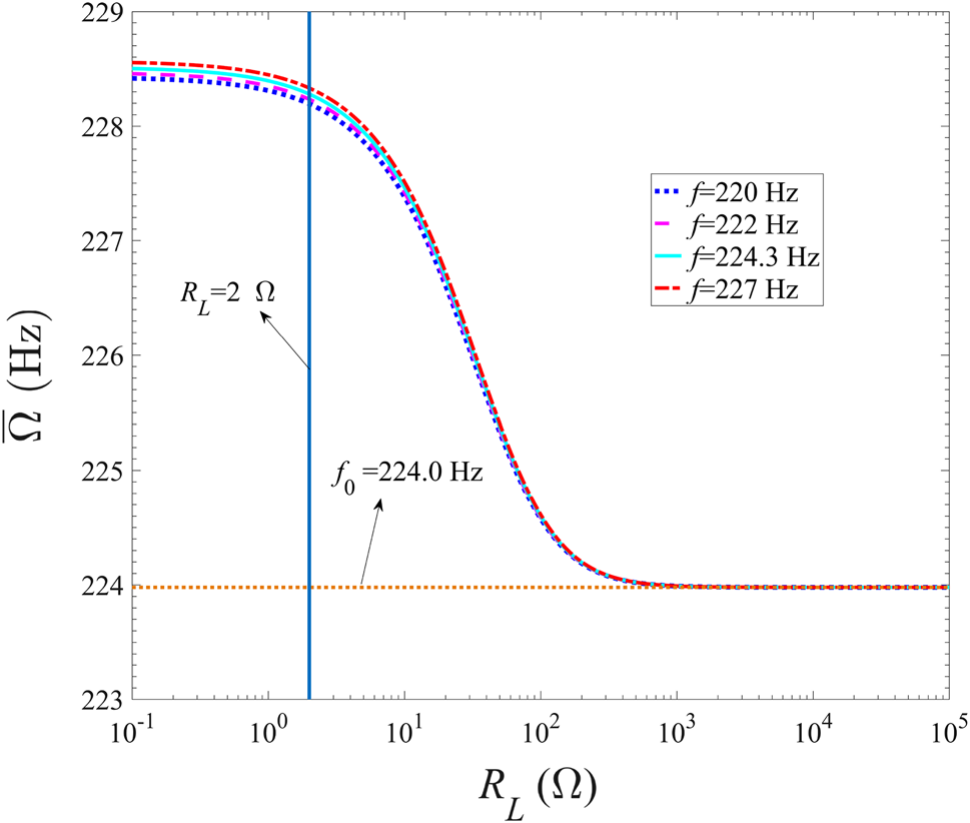
Variations of modified frequency with the external resistance for different excitation frequencies.

Figure 5 shows the variations of the modified frequency of the energy harvester with the external resistance. As shown in Fig. 5, with the gradual increase of external resistance, the modified frequency gradually decreases, and tends to the first-order natural frequency of the model when the resistance is greater than $10^3$ $\Omega$. Similar to the characterization of the variation of electrical damping with resistance, the decreasing trend is weak when the resistance is less than 2 $\Omega$ and relatively strong when the resistance is between 2 $\Omega$ and $10^3$ $\Omega$. Besides, as the excitation frequency increases, the modified frequency of the energy harvesting model increases. However, when the resistance is greater than $10^3$ $\Omega$, the modified frequency is basically the same, which is equal to the natural frequency of the energy-harvested model. Therefore, the electromechanical coupling effects of the coupled Galfenol energy harvester become weaker with the resistance increases, as same as the result reported by Khazaee^26^ that greater $\frac{{\bar{\Omega }}}{\omega }$ means greater electromechanical coupling effects for the piezoelectric harvesters.

**Figure 6:**
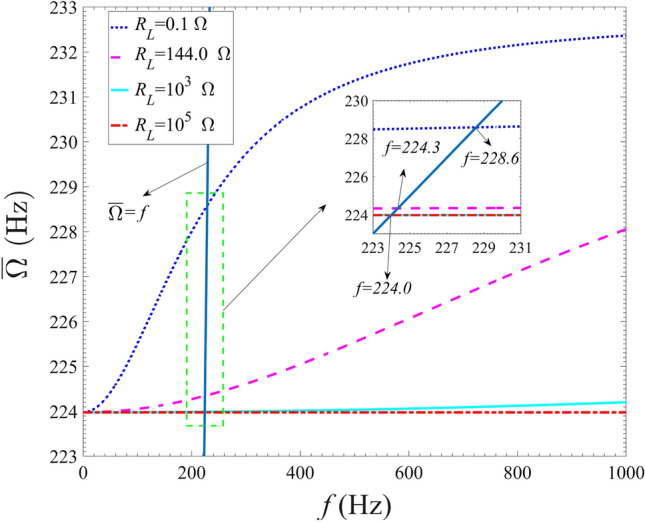
Variations of modified frequency with the excitation frequency for different external load resistances.

The variations of the modified frequency of the energy harvester model with the excitation frequency are presented in Fig. 6. As shown in Fig. 6, with the gradual increase of external resistance, the modified frequency gradually decreases. It can be seen that the modified frequency gradually increases from 224.0 Hz with the increase of excitation frequency for ${R_L} = 0.1$ $\Omega$ , ${R_L} = 144.0$ $\Omega$ and ${R_L} = {10^3}$ $\Omega$. When the external resistance is set as ${R_L} = {10^5}$ $\Omega$ , the modified frequency remains basically unchanged as the excitation frequency increases. In general, similar to electric damping, when the resistance is larger than $10^3$ $\Omega$ , the modified frequency is no longer sensitive to the excitation frequency. Unlike the trend of electric damping changing with excitation frequency, the maximum change rate of the modified frequency on the excitation frequency is 3.75$\%$ for the four discussed external resistances. In other words, the effect of the external excitation frequency on the increase of coupling frequency ($\frac{{{C_g}{\theta _g}^2{\omega _b}^2}}{{R\left( {1 + {C_g}^2{\omega _b}^2} \right) }}$) is relatively small. Although this coupling frequency variation seems small, it can have a substantial effect on the output performances, as the output power is narrowband due to the high electromechanical coupling change rate of the energy harvester over the resonance region. In addition, when the condition that the modified frequency is equal to the excitation frequency is satisfied, the corresponding excitation frequency is about 224 Hz for ${R_L} = {10^3}$ $\Omega$ and ${R_L} = {10^5}$ $\Omega$, 224.3 Hz for ${R_L} = 144.0$ $\Omega$, and 228.6 Hz for ${R_L} = {0.1}$ $\Omega$.

#### Effects of external load resistance on the output responses

**Figure 7:**
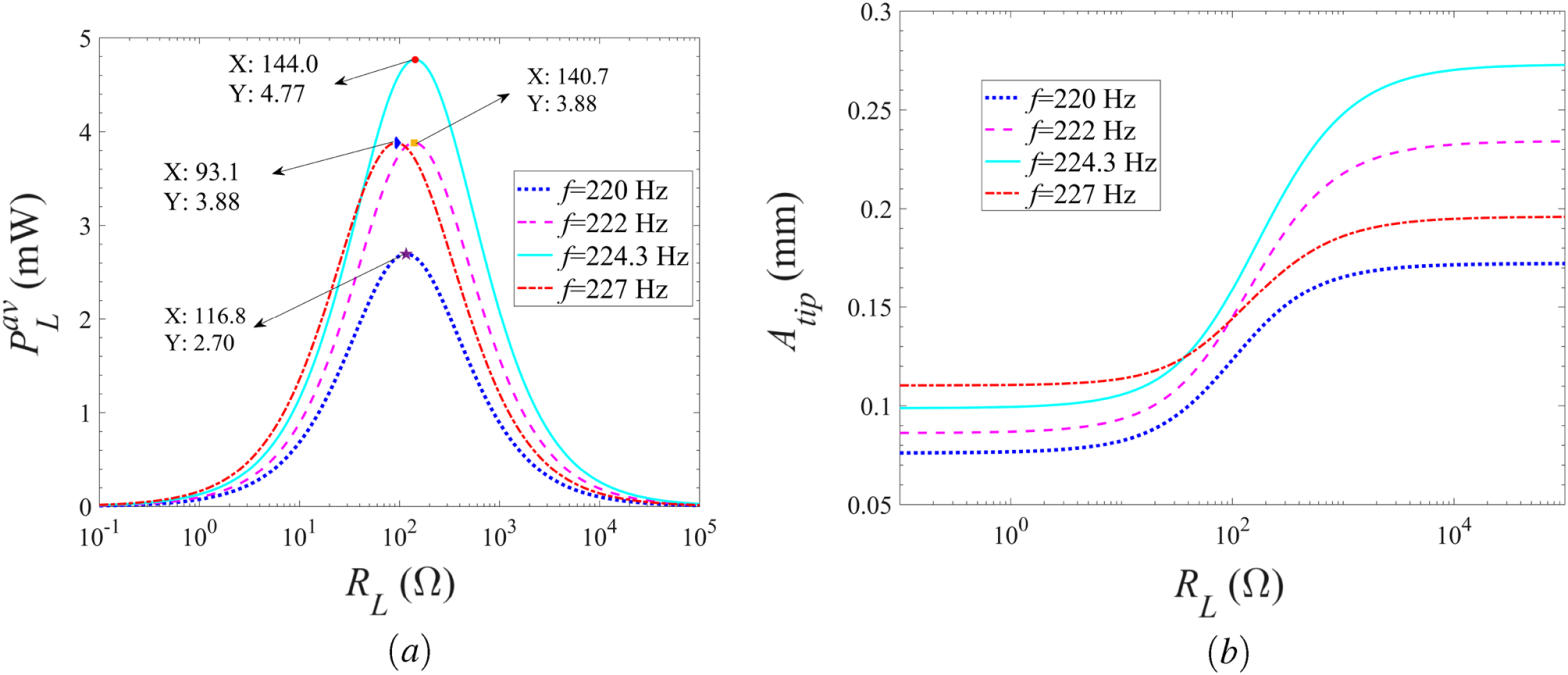
Variations of the (**a**) average harvested power and (**b**) vibration displacement of the energy harvester with the external load resistance for different excitation frequencies.

Figure 7 shows the average harvested power and vibration displacement of the Galfenol energy harvester with the external resistance at different excitation frequencies for the acceleration amplitude of ${a_0} = 15$ $\mathrm m/{s^2}$. From Fig. 7a, it can be seen that with the increase of the external resistance, the average harvested power of the energy harvester increases, then decreases, and finally tends to zero for all considered excitation frequencies. Thus, there is an optimal external load resistance, which makes the energy harvester obtain the maximum harvested power. In addition, as the excitation frequency increases, the maximum average output power and the corresponding optimal external load resistance of the energy harvester rise first and then decrease. The maximum harvested power is achieved when the excitation frequency is set as 224.3 Hz, and the corresponding optimal resistance is 144.0 $\Omega$. Referring to Fig. 7b, with the increase of external load resistance, the vibration displacement of the energy harvested firstly keeps steady for ${R_L} < {10^1}$ $\Omega$, then gradually rises for ${10^1}$ $\Omega \le {R_L} \le {10^4}$ $\Omega$, and finally tends to be steady for ${R_L} > {10^4}$ $\Omega$. Obviously, the load resistance range corresponding to the significant increase of vibration displacement is consistent with that corresponding to the drastic change of harvested power. This is mainly because the resonance response of the energy harvesting model is excited within this load resistance range which is defined as the resonance region for the load resistance. Additionally, the mentioned resonance region for the energy harvester first increases and then decreases as the excitation frequency increases. Obviously, the output performances exhibit a strong dependence on the load resistance within the resonance region where the change rate of the electromechanical coupling properties is drastic. In general, stronger dependence of the electrical damping on the load resistance means greater electromechanical coupling effects on the output performances.

**Figure 8:**
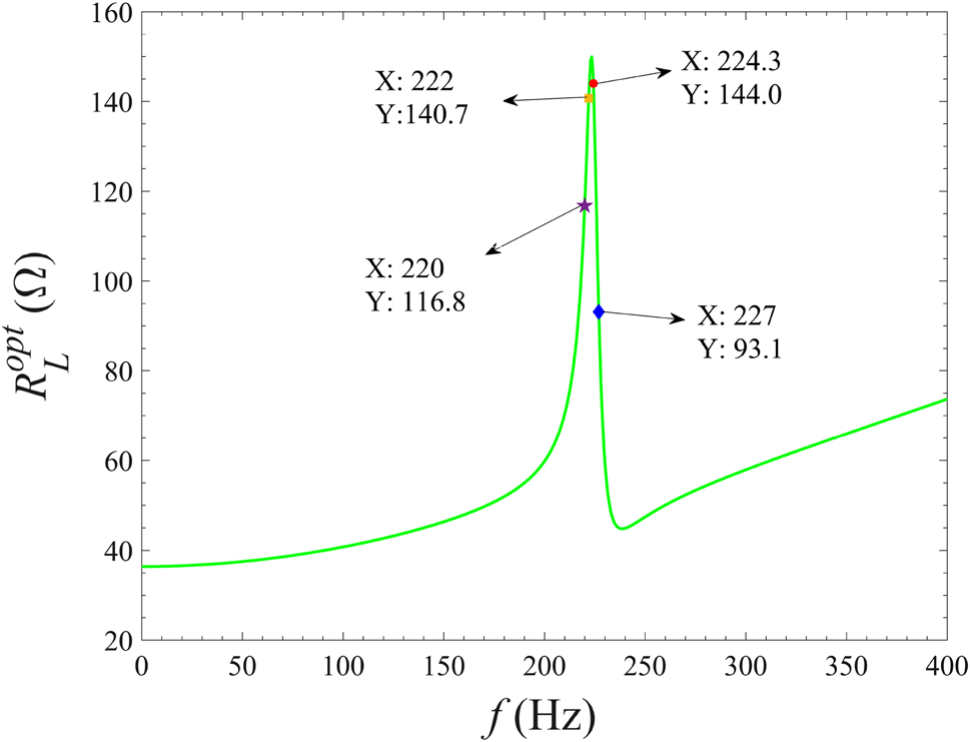
Variation of the optimal external resistance with the excitation frequency.

To investigate the relationship between the optimal resistance and the external excitation frequency, the optimal external load resistance $R_L^{opt}$ is calculated by Eq. (26) and the calculation results are shown in Fig. 8. As illustrated from Fig. 8, it is obvious that the optimal external resistance calculated by Eq. (26) is consistent with the results illustrated in the Fig. 7a. With the increase of the excitation frequency, the optimal external resistance shows a trend of first increasing, then decreasing and gradually increasing. Obviously, the varying trend of the optimal resistance is monotonous, that is, once the excitation frequency is fixed, there only one optimal resistance exists to achieve the maximum harvested power.

#### Effects of excitation frequency on the output responses

**Figure 9:**
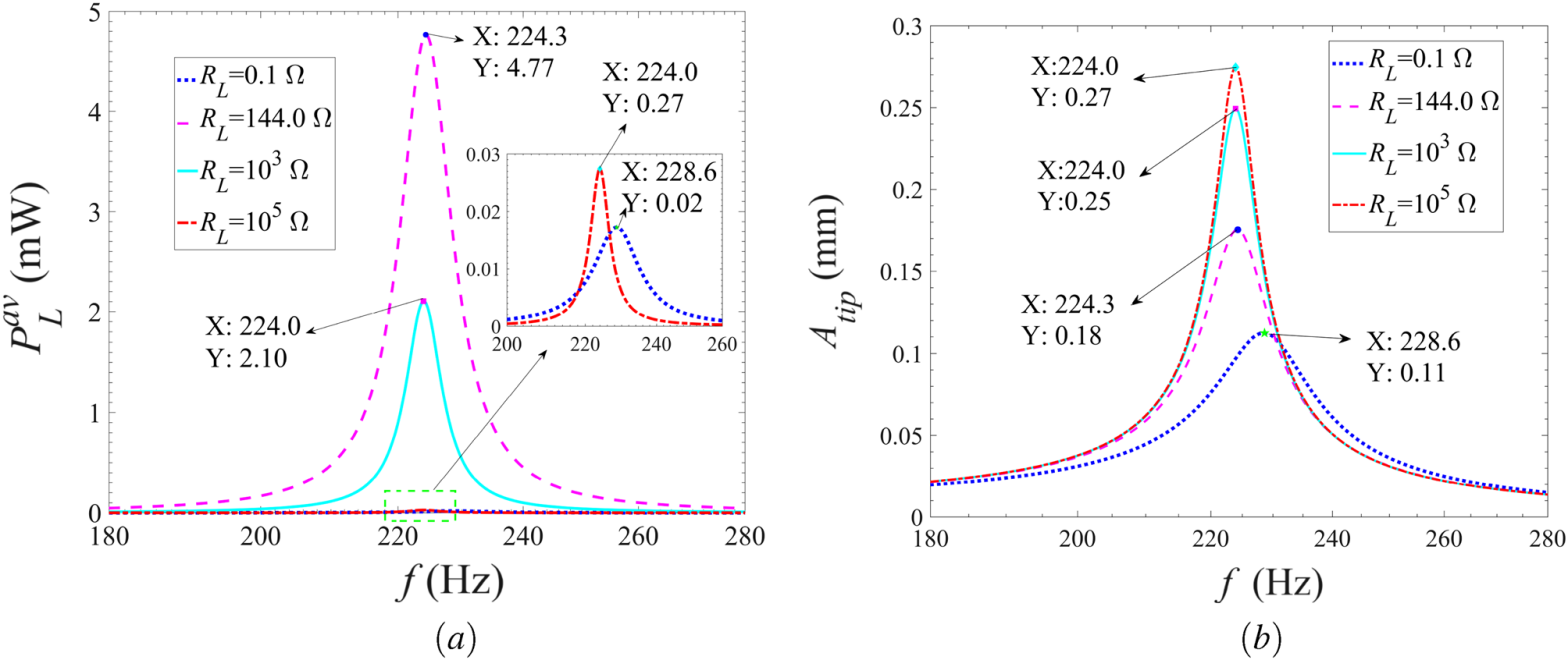
Variations of the (**a**) average harvested power and (**b**) vibration displacement of the energy harvester with the excitation frequency for different external load resistances.

Figure 9 shows the variations of the average harvested power of the Galfenol energy harvester with the excitation frequency for different external resistances at the external excitation acceleration amplitude of ${a_0} = 15$ $\mathrm m/{s^2}$. Referring to Fig. 9, as the excitation frequency increases, the average harvested power and the vibration displacement of the energy harvester increase firstly, then decrease and finally tend to zero for all discussed external resistances. Therefore, an optimal excitation frequency exists to obtain the maximum harvested power. Fig. 9a shows that, with the increase of the external load resistance, the maximum average output power of the energy harvester increases first and then decreases. While the corresponding optimal excitation frequency is decreasing as the external load resistance increases. The maximum harvested power is obtained with the external load resistance set as 144.0 $\Omega$ , and the corresponding optimal excitation frequency is 224.3 Hz. Obviously, the optimal result is corresponding to that illustrated in Fig. 7a. As shown in Fig. 9b, the maximum vibration displacement and corresponding excitation frequency of the energy harvester increases and decreases respectively as the external load resistance increases. For the case of ${R_L} = 144.0$ $\Omega$, the maximum harvested energy is achieved with relatively small vibration displacement at $f = 224.3$ $\mathrm{{ Hz}}$, which is the expected result. It is noted that, for all considered external load resistances, the optimal excitation frequency is equal to the results calculated by solving the equation of ${\bar{\Omega }^2} - \omega _b^2 = 0$, as illustrated in Figs. 6 and 9a. The reason is that when the modified frequency $\bar{\Omega }$ is equal to the excitation frequency $\omega _b$, the strongest resonance response occurs. Moreover, better harvesting energy performance will manifest itself when the external load resistances are in the resonance region for the greater electromechanical coupling effects.

**Figure 10:**
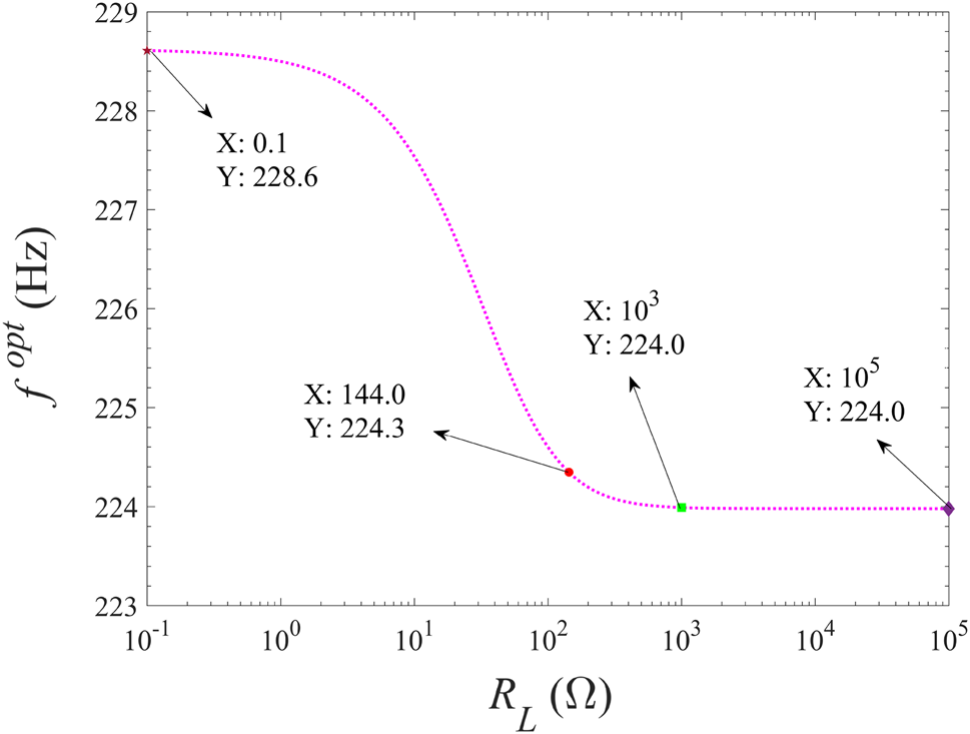
Variation of optimal excitation frequency with external load resistance.

In order to study the variation of the optimal excitation frequency with the external load resistance, calculate the optimal excitation frequency ${f^{opt}}$ by Eq. (27) and plot the calculation results in Fig. 10. As can be seen in Fig. 10, the optimal excitation frequency obtained from Eq. (27) is completely consistent with the results shown in Fig. 9. That is, when the external load resistance is fixed, the optimal excitation frequency can be obtained by Eq. (27) as well as the equation given by ${\bar{\Omega }^2} - \omega _b^2 = 0$. In addition, with the increase of external resistance, the optimal excitation frequency decreases monotonically. Therefore, there is always an optimal excitation frequency to make the energy harvester obtain the maximum harvested power.

## Conclusions

The vibration energy harvesting based on the magnetostrictive mechanism provides a new type of method for energy harvesting. In this work, the electromechanical coupled distributed parameter model of the Galfenol cantilever energy harvester is established using Hamilton’s principle, linear constitutive equations of magnetostrictive material and Faraday’s law of electromagnetic induction. The explicit analytical solutions of average harvested power across the load resistance and tip vibration displacement of the Galfenol energy harvester are then derived as functions of the mechanical, circuit and external excitation parameters by utilizing the methods of Galliakin decomposition and the electromechanical decoupling. The proposed analytical solutions of the output response of the harvester show good agreement with the numerical solutions^41^ and are basically consistent with the experimental data^40^. The analytical solutions of the harvesters revealed that, for any fixed excitation frequency or external load resistance, the corresponding optimal external load resistance or excitation frequency could be found to achieve the maximum harvested power from the related exact relationship between the output performances and the electromechanical coupling parameter at any excitation frequency and any external load resistance.

In general, the electromechanical coupling alters the natural frequency and the damping of the cantilever beam and thus affects its mechanical displacement and harvested power of the Galfenol energy harvester. We suggest estimating the mechanical displacement and harvested power by accounting for the change in the natural frequency and damping instead of solving the coupled equations. The proposed decoupled model provides a simplified approach to account for the electromechanical coupling and gives satisfying results for the engineering application of cantilever-beam Galfenol energy harvesters, which will significantly facilitate the optimal analyzation and the modulations between the electrical and mechanical for the Galfenol energy harvester.

## Data Availability

The datasets used and analysed during the current study available from the corresponding author on reasonable request.
